# Marine Bacterial Ribosomal Peptides: Recent Genomics- and Synthetic Biology-Based Discoveries and Biosynthetic Studies

**DOI:** 10.3390/md20090544

**Published:** 2022-08-24

**Authors:** Linda Sukmarini

**Affiliations:** Research Center for Applied Microbiology, National Research and Innovation Agency (BRIN), Jl. Raya Bogor, Km. 46, Cibinong 16911, West Java, Indonesia; linda.sukmarini@brin.go.id

**Keywords:** marine bacteria, ribosomal peptides/RiPPs, genomics, genome mining, synthetic biology, biosynthetic gene clusters

## Abstract

Marine biodiversity is represented by an exceptional and ample array of intriguing natural product chemistries. Due to their extensive post-translational modifications, ribosomal peptides—also known as ribosomally synthesized and post-translationally modified peptides (RiPPs)—exemplify a widely diverse class of natural products, endowing a broad range of pharmaceutically and biotechnologically relevant properties for therapeutic or industrial applications. Most RiPPs are of bacterial origin, yet their marine derivatives have been quite rarely investigated. Given the rapid advancement engaged in a more powerful genomics approach, more biosynthetic gene clusters and pathways for these ribosomal peptides continue to be increasingly characterized. Moreover, the genome-mining approach in integration with synthetic biology techniques has markedly led to a revolution of RiPP natural product discovery. Therefore, this present short review article focuses on the recent discovery of RiPPs from marine bacteria based on genome mining and synthetic biology approaches during the past decade. Their biosynthetic studies are discussed herein, particularly the organization of targeted biosynthetic gene clusters linked to the encoded RiPPs with potential bioactivities.

## 1. Introduction

The marine environment encompasses up to 71% of the Earth’s surface, with an incredibly rich biosphere of flora and fauna—including plant and animal species as well as a vast number of microbes [1,2,3]. Marine microbes have been found to inhabit all parts of the marine environment, including seawater, sediments, and association/symbiosis with marine macro-organisms [4,5,6]. They have physiological, biochemical, and metabolic capabilities that enable them to survive in their unique habitats by producing defensive secondary metabolites of marine natural products. Given its tremendous wealth of microbial diversity, the marine biosphere offers an excellent opportunity for the discovery and development of microbial-derived pharmacologically active compounds or other potentially bioactive compounds of biotechnological or commercial value [7,8,9,10].

Marine bioactive natural products represent various compound classes ranging from fatty acids, terpenoids, and polyketides to peptides [11]. Moreover, peptides have been regarded as important bioactive natural products with vast and diverse chemical spaces. Ribosomal peptides are a class of peptide-based natural products that are subjected to extensive post-translational modifications (PTMs) to generate structurally diverse bioactive peptides. Such peptide natural products are so-called ribosomally synthesized and post-translationally modified peptides (RiPPs). Due to the extensive PTMs in these peptides, they are able to attain a similar degree to their counterpart’s—non-ribosomal peptides—chemical diversity that endows them with a broad range of potent biological activities [12]. Many RiPPs have been reported synthesized from fungi, plants, and metazoans [13,14,15], and they were predominantly discovered from bacteria [16]. However, marine bacteria are still less explored sources of RiPPs—apart from the predominant example of marine cyanobacterial cyanobactins. Cyanobactin patellamides A and C were the first example of RiPPs product biosynthesis from marine symbiotic cyanobacteria, *Prochloron didemni*, which were initially isolated from ascidian *Lissoclinum patella* [17]. Patellins and trunkamides of cyanobactins were then also found to be synthesized from *Prochloron* spp. [18]. Of note, the discovery of these cyanobactins’ biosynthesis has been driven by genome-scale sequence information [19,20,21,22].

**Figure 1:**
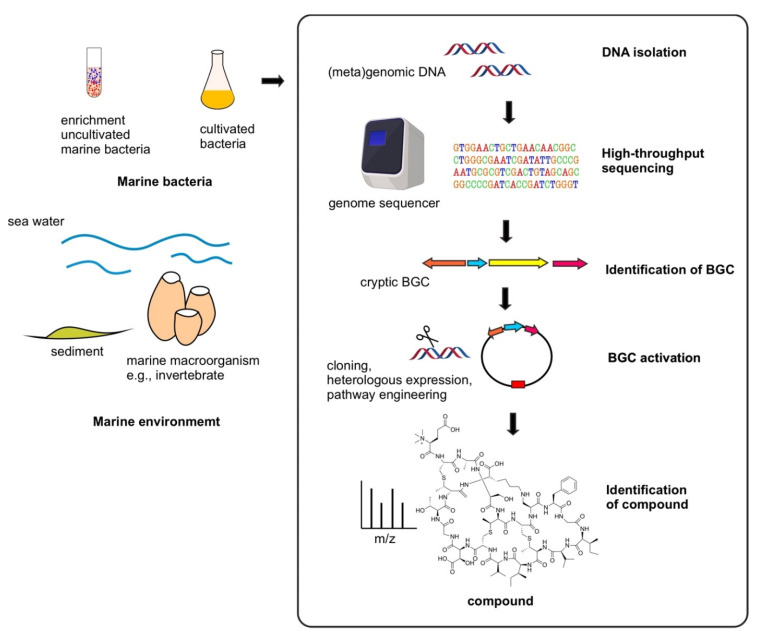
General schematic of genomics and synthetic biology-based discovery of marine bacterial RiPPs.

Increased knowledge of RiPP natural product biosynthesis has coupled with the advancement of next-generation DNA sequencing technology and bioinformatics tools in the postgenomic era [23,24]. As genome sequence information is rapidly and increasingly retrieved, it has led to the development of microbial genome mining approaches—including metagenomics for obtaining the uncultivated bacteria—to isolate previously unprecedented cryptic microbial natural product biosynthetic gene clusters (BGCs) in the genome, accompanied by various BGC activation methods to envisage the compound synthesis. Notably, high uniqueness and novelty potential are present for RiPPs with unusual BGCs that are frequently unrecognized in genomes. An integrated strategy of genome mining and synthetic biology toward the BGC awakening, in vivo heterologous expression systems and pathway engineering have expedited the progress of microbial natural product discovery and development (Figure 1) [25,26,27,28].

Recent extensive previous reviews have discussed RiPPs from bacteria (proteusin RiPPs) [29], fungi-sourced biosynthetic pathways [14], and eukaryotic, including plant- and animal-derived RiPPs [13]. Moreover, their enzymology, bioengineering and applications have also been broadly reviewed elsewhere [30,31,32,33]. This present short review, however, provides an overview of the recent updates of the discovery of RiPPs from marine bacteria employing genome mining and synthetic biology approaches during the past decade (2012–2022) with an emphasis on their biosynthetic studies.

## 2. General Biosynthesis of Bacterial RiPPs

**Figure 2:**
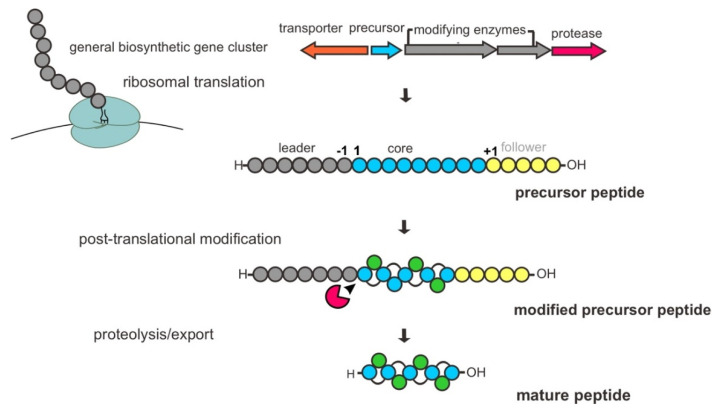
General schematic of the biosynthetic pathway of bacterial RiPPs.

The biosynthetic pathway of RiPPs is encoded in the genome by BGCs, typically comprising one or several genes encoding for precursor peptides, PTM modifying enzymes, proteases and a transporter protein. As mentioned earlier, they are defined by a common biosynthetic feature of the enzymatic PTM process. Basically, a bacterial RiPP biosynthesis begins with the ribosomal synthesis of an inactive gene-encoded linear precursor peptide, usually 20–110 amino acids, composed of an N-terminal leader peptide sequence and C-terminal core peptide sequence with optional signal and recognition/follower peptide sequences. The specific motifs within the leader peptide are recognized and bound by dedicated PTM enzymes that lead to the processing or decoration of the peptide in the core region. Subsequently, the modified peptide undergoes proteolytic cleavage of the leader peptide by protease(s), and additional PTMs are present in certain cases for further tailoring of the modified peptide. Finally, the mature peptide is released from cells as a bioactive peptide through a transporter protein, such as a member of the adenosine triphosphate (ATP)-binding cassette (ABC) transporter family (Figure 2) [12,34,35,36].

Although RiPPs synthesized from the ribosome depend on a set of 20 standard canonical or proteinogenic amino acids, the PTMs involved in their biosynthesis are assorted, e.g., dehydration, cyclization, hydroxylation, methylation, epimerization, phosphorylation, and cross-linked disulfides, generating a vast array of structures. The installation of these diverse chemical functionalities confers important biological properties, e.g., the stability of substrate bioactivities [12]. Moreover, the modifying enzymes have demonstrated a high degree of substrate promiscuity with regard to precursor core peptide sequences, featuring the potential significance of synthetic biology applications generating novel RiPP variants with appealing properties [28].

Owing to the remarkable diversity of novel PTMs discovered over the past decade, more, new RiPP classes and their representative examples have been notably introduced [31]. Herein, RiPPs derived from marine bacteria are summarized in Table 1.

## 3. Proteusin-Derived Polytheonamides

Polytheonamides, polytheonamides A and B—known as potent natural product cytotoxins—were initially considered non-ribosomal peptides with origin from the marine sponge *Theonella swinhoei* as they have structural intricacy, including multiple d-configuration amino acids and N-and C-methylations [42]. However, a metagenomic study of this microbially rich sponge has unveiled a bacterial biosynthesis gene design. The mining of BGC for polytheonamide synthesis supported that these peptides were generated through a ribosomal pathway. Moreover, assisted by the single-cell analysis of the microbiome of *T. swinhoei*, it was shown that polytheonamides are genuinely derived from the endosymbiont *Candidatus* Entotheonella factor [37,43,44]. Given the series of codon sequences conforming exactly to a complete polytheonamide precursor and immensely variable modifying enzymes, polytheonamides belong to the RiPP class of proteusins. This class is characterized by a typical precursor structure highlighting an unusual leader peptide with homology to leader peptides of nitrile hydratases—named nitrile hydratase-like leader peptides (NHLPs)—rather than defining PTMs due to the lack of similar enzymology [31]. Interestingly, most peptides that belong to this class are genome-predicted products [29].

**Figure 3:**
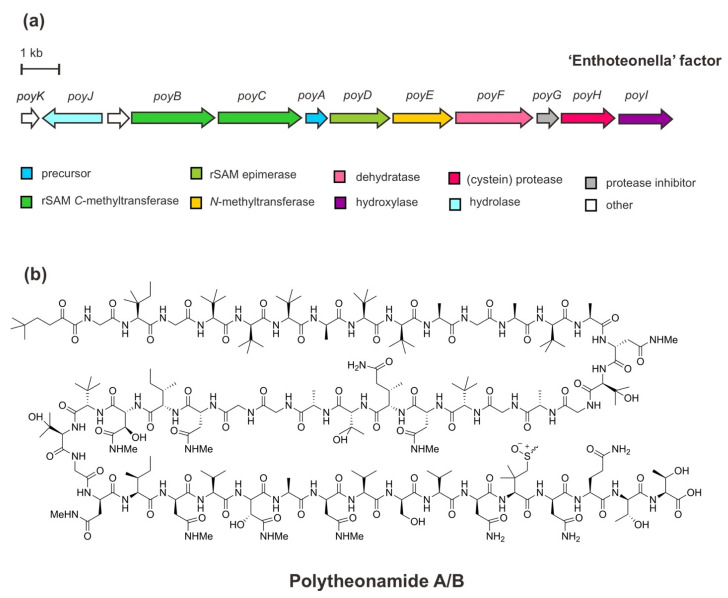
(**a**) Schematic architecture of the biosynthetic gene cluster of polytheonamides and (**b**) the chemical structures of polytheonamides A and B differ in the configuration of sulfoxide moiety.

The BGC of polytheonamides (Figure 3) comprises a structural gene (*PoyA*), genes of the PTM machinery (*PoyBCD*, *PoyE*, *PoyI*, and *PoyF*), a proteolytic gene (*PoyH*), a regulator/protease inhibitor gene (*poyG*), a putative membrane/transport-hydrolase gene (*PoyI*), and an as yet an unknown function gene *PoyK*. The structural gene *PoyA* encodes the linear precursor peptide *PoyA* and contains unusual NHLP leader sequence as recognition for modifying enzymes to undergo the extensive PTMs of core polytheonamides in the C-terminal region. Functional studies via heterologous co-expression in *Escherichia coli* have enabled the characterization of those enzymes responsible for diverse PTMs, including epimerizations, methylations, and hydroxylations during the maturation process. In brief, through iterative and irreversible l-to-d epimerizations carried out by a single radical *S*-adenosylmethionine (rSAM) enzyme, *PoyD*, 18 d-amino acid residues are introduced. Moreover, eight d-configurated-epimerized asparagine (Asn) residues are iteratively N-methylated by the SAM-dependent N-methyltransferase enzyme, *PoyE*. While, 17 C-methylations occurred at unactivated carbons of diverse amino acid residues catalyzed by *PoyB* and *PoyC*, the putative cobalamin-dependent rSAM C-methyltransferases. Four hydroxylation reactions of valine and N-methyl Asn residues are catalyzed by a putative Fe(II)/α-ketoglutarate oxidoreductase enzyme, *PoyI*. In addition, four methylations of an N-terminal threonine (Thr) residue are also installed by *PoyC*, along with single dehydration at this residue by *PoyF*—a dehydratase domain of LanM-type lantibiotic synthetase enzyme—leads to an unusual *t*-butylated N-acyl moiety. Subsequent to the PTMs, the proteolytic cleavage by the cysteine protease *PoyH* releases the core peptides and elicits hydrolysis of an N-terminal enamine at the *t*-butylated Thr to the α-keto moiety of polytheonamides [44,45,46,47,48]. Presumably, mature active polytheonamides are formed.

In addition to the aforementioned genes encoding proteins involved in polytheonamide processing, *PoyG* has been shown to inhibit *PoyH* cleavage. This inhibitor protein seems to control the proteolytic activity of *PoyH* in the maturation process of polytheonamides. Likely, both *PoyH* and *PoyG* proteins are encoded in the same transcript, but a discrepancy in their regulation exists during translation or PTM processing [47]. Furthermore, although *PoyJ* showed similarity to prolyl oligopeptidases—a class of enzyme involved in the peptide cleavage instead of the export event—it did not cleave *PoyA* in vivo [46]. However, another putative function of this protein as a membrane transport protein with an α/β hydrolase fold domain needs further study. Moreover, *PoyK* is not involved in the biosynthesis of the peptides, and its function remains elusive.

To date, polytheonamides remain the largest and most complex RiPPs from the uncultivated marine symbiont due to their very extensive PTM processing. The installation of these complex enzymes has been shown to have important functions in the biological properties of the peptides—particularly the cytotoxicity of polytheonamide B—and their derivatives [49,50,51]. The N-terminal functionality, as well as the hydrophobicity of the peptide, have been demonstrated to contribute to increased membrane penetration and pore-generating activity, which led to considerable cytotoxicity. Additionally, the introduction of d-configurated residues forces the peptide to maintain a β-helical structure and impart resistance to proteolysis. The stability of its nanotube β-helical structure is also preserved through N-methylations of hydrogen bond clamps within the helix conformation [44,52]. Polytheonamide B has also been reported to have an antibacterial effect through depolarization of the bacterial cytoplasmic membrane [37].

## 4. Ammosamides and Lymphostin of a New Class of Pearlins

Known as bacterial pyrroloquinoline alkaloids, potent cytotoxic ammosamides and immunosuppressant lymphostin now belong to the class of recently discovered peptide aminoacyl-tRNA ligases (PEARLs or pearlins). The unifying feature of this class is the primary aminoacyl-tRNA-dependent extension of a ribosomal peptide natural product. Basically, pearlins utilize RiPP as a scaffold to extend non-ribosomal peptides and chemical modification. Following the peptide extension, the attached residue is subsequently subjected to further modification, while the first formed peptide bond endures proteolysis at the end biosynthetic pathway. Genome information revealed that the enzymes share homology to LanB dehydratases of lanthipeptide biosynthesis featuring the glutamylation domain but lacking the elimination domain—named small LanB (sLanB) [31,53].

**Figure 4:**
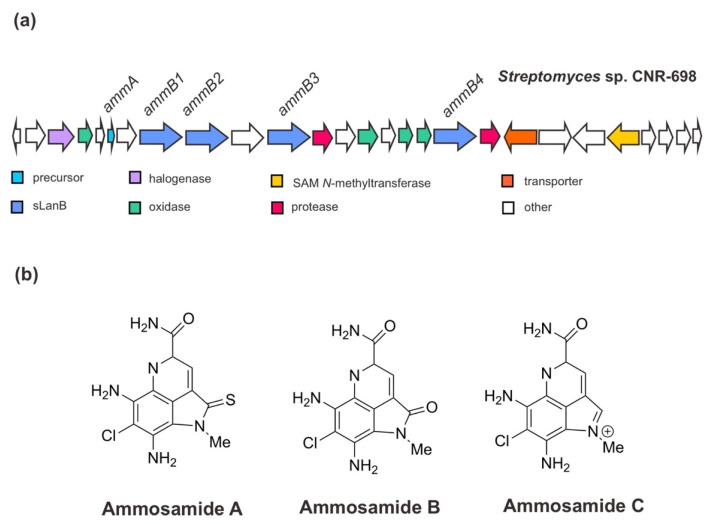
(**a**) Schematic architecture of the biosynthetic gene cluster of ammosamides and (**b**) the chemical structures of ammosamides A–C. The Figure is only schematic and not to approximate scale.

Initially, Jordan and Moore (2016) [38] ascertained the BGC for ammosamides A–C from *Streptomyces* sp. CNR-698 [54]. The genomic study of this deep-marine sediment strain revealed that the gene cluster for ammosamide biosynthesis consists of 27 open reading frames (ORFs). Assisted by the genetic manipulation and heterologous expression especially of the orphan genes, the ammosamides’ BGC has been corroborated and it was shown that these small molecules are originated from a complex set of non-canonical biosynthetic pathways associated with RiPP biosynthetic genes. The BGC of ammosamides (Figure 4) encodes a precursor peptide AmmA; the PTM enzymes, especially four sLanB dehydratases (AmmB1, AmmB2, AmmB3, AmmB4), a MibH-like flavin-dependent tryptophan (Trp) halogenase, and flavin-dependent oxidoreductase proteins; two proteases; a transporter protein and other hypothetical proteins [38,53,55]. In addition, it has been suggested that the modifications of the pyrroloquinoline core, such as oxidative primary amide bond formation and N-methylation catalyzed by a putative coenzyme F420-dependent oxidase (Amm4) and a putative SAM-dependent methyltransferase (Amm23), respectively, occurs at the late stage of biosynthesis. The chlorination of Trp is likely an early event with a specific Trp substrate since the product was completely abolished in the absence of the Trp halogenase gene (previously annotated *amm3*), and it was not rescued by supplementation with 6-Cl-Trp [38].

In view of the biosynthesis of a small non-ribosomally synthesized molecule 3-thioglutamate employing a glutamyl-tRNA-dependent mechanism of RiPP biosynthesis for their extension assembly, a further study on the BGC of ammosamides by van der Donk and his co-workers (2019) [53] has given a new insight into the role of sLanB and the scaffold peptide. Contrary to the previous indication [38], the recent finding has demonstrated that AmmA is likely to have a C-terminal extension of an additional Trp residue instead of it being embedded in a C-terminal Trp (encoded in the gene cluster). One of sLanB, AmmB2—not the other AmmB1, AmmB3, and AmmB4 proteins—has been found to add a Trp residue to the C-terminus of a truncated peptide of AmmA in vitro and in vivo in *E. coli* in a tRNA^Trp^ and a Trp-RS-dependent manner. The mutation or deletion of the C-terminal Trp remained functional for peptide to restore the peptide production, accounting for these peptides produced when the C-terminal Trp was eliminated [53]. Eventually, it became apparent that proteolysis of a terminal Trp yields ammosamides. This finding was also in agreement with the observation made for the catalytic role of the scaffold peptide, where ammosamide C is the dominant pathway product requiring the successive formation of either AmmA or AmmB [38].

**Figure 5:**
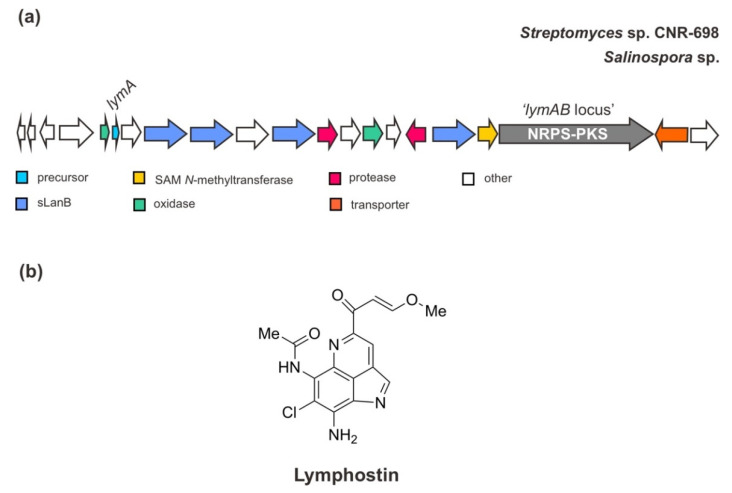
(**a**) Schematic architecture of the biosynthetic gene cluster of lymphostin and (**b**) the chemical structure of lymphostin. The Figure is only schematic and not to approximate scale.

As mentioned above, the BGC of ammosamides is also similar to the biosynthetic pathway of another closely related pyrroloquinoline alkaloid, lymphostin [38] (Figure 5). Interestingly, through genome sequencing, the gene cluster for lymphostin biosynthesis has also been identified in other marine strains of the genus *Salinospora—S. tropica* CNB-440, *S. aeronicola* CNS-205, and *S. pacifica*. Ultimately, this compound utilizes a similar mechanism of non-ribosomal peptide-polyketide (NRPS-PKS hybrid) extension during RiPP biosynthesis, generating a joint structure of RiPP and PKS. The assembly line of its PKS hybrid has been well described previously [56].

## 5. A Cinnamycin-like Lanthipeptide: Divamide A

As mentioned previously, metagenome sequencing has been regarded as a powerful method to access uncultivated bacteria as a reservoir for novel cryptic BGCs connected to important bioactive natural products. Moreover, the application of this method to confirm chemical structures due to limited source supply has also been demonstrated by the discovery of a novel lanthipeptide structure of divamides [39].

Originally, divamide A—a potent anti-HIV agent—was extracted and characterized from marine tunicate *Didemnum molle* E11–036 collected from the coral reef in Papua New Guinea. The structure of divamide A was determined by means of nuclear magnetic resonance (NMR) spectroscopy combined with metagenomic data analysis and mass spectrometric (MS^2^) fragmentation [39]. It is suggested that divamide A belongs to RiPPs related to the class II lanthipeptide cinnamycin produced by *Streptomyces cinnamoneous* [57]. Divamide A features three methyllanthionines (MeLan), a lysinolanine (Lal), a β-hydroxy-aspartate (Asp) residue, and a rare N-trimethylated glutamate (Glu) residue. However, in the search for a candidate precursor peptide in the tunicate metagenome, the BGC was verified in the uncultivated symbiotic cyanobacterium *Prochloron didemni* confirming the peptide is derived from bacterial origin.

**Figure 6:**
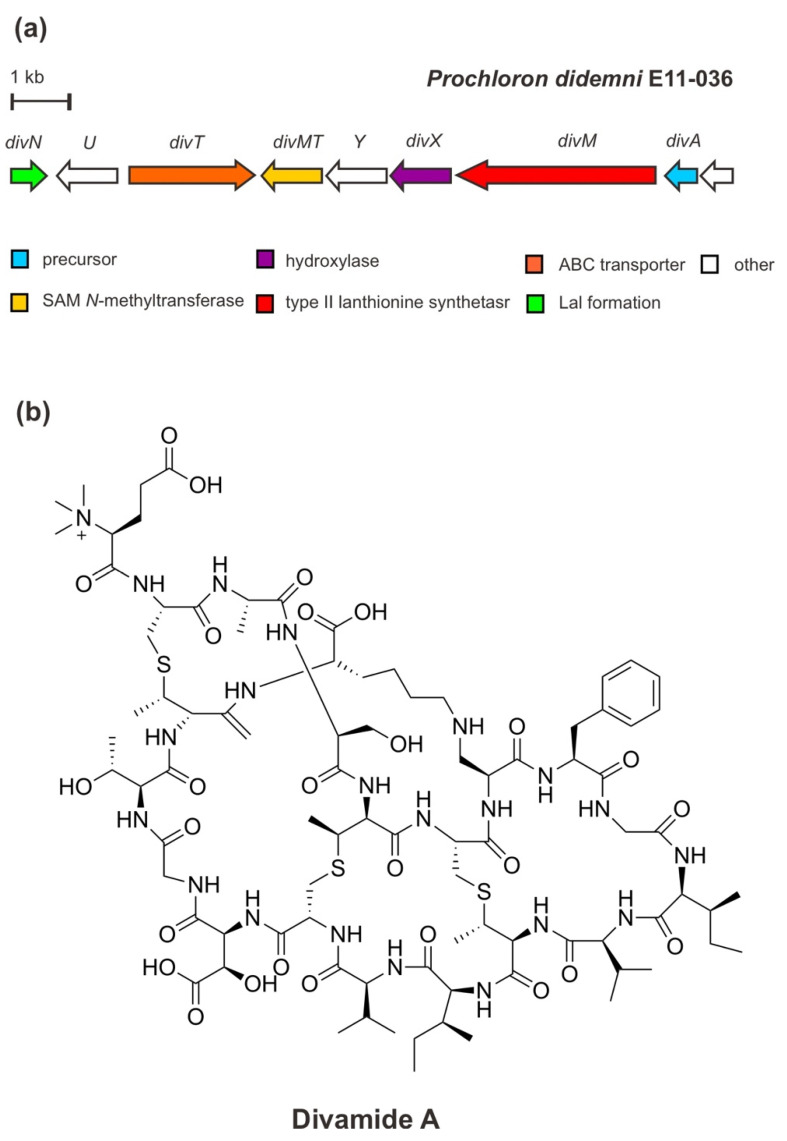
(**a**) Schematic architecture of the biosynthetic gene cluster of divamide A and (**b**) the chemical structure of divamide A.

The metagenomic study of P. didemni [39] revealed that the BGC of divamide A (Figure 6) consists of a structural precursor gene (divA), the PTM genes (divM, divX, divN, divMT), an ABC-type transporter gene (divT), and two genes of unknown function. The structural gene divA encodes the precursor peptide, containing cysteine (Cys), serine (Ser), threonine (Thr) residues in the core peptide of the C-terminal precursor peptide, as well as the sequence “GTTK” motif (glutamic acid (Glu)/threonine (Thr)/threonine (Thr)/lysin (Lys)). Cys/Ser/Thr is modified by the PTM enzyme DivM—a type II lanthionine synthetase carrying an N-terminal dehydratase and a C-terminal cyclase domain— to generate the unusual amino acids dehydroalanine (Dha) and dehydrobutyrine leading to the installation of three MeLan cross-links. These cross-links are the biosynthetic feature of lanthipeptides. In addition to this class-defining lanthionine, other PTM installations include Asp hydroxylation by an α-ketoglutarate-dependent Fe (II) monooxygenase/β-Asp hydroxylase, DivX, generating a β-hydroxy-aspartate (Asp) residue; lysinoalanine cross-link formation via the addition of lysine to a Dha catalyzed by DivN (a homolog of DurN protein in duramycin biosynthesis); and N-terminal trimethylation by a SAM-dependent methyltransferase, DivMT. Trimethylation of an N-terminal residue is currently a unique PTM in RiPPs. [31,39]. Moreover, an intact BGC of divamide A was reconstituted and expressed in the heterologous E. coli cell and yielded an unmethylated precursor lacking the lysinoalanine residue. This feature, however, was subsequently introduced by the chemical treatment with a certain alkaline condition [58]. Lysinoalanine was shown to be critical for the activity of DivMT in vitro, catalyzing N-terminal trimethylation. It has been suggested that in each pathway, the methyl moieties are introduced by a single SAM-dependent methyltransferase in a final maturation event following the proteolytic core peptide release [31,39].

It is important to note that the macrocyclic or cross-link feature, including MeLan and lysinoalanine, lead to conformational rigidity that confers target binding and prevents or minimizes proteolysis [12,31]. The synthetic biology approach has determined the structure–activity relationship (SAR) to be considered for further mechanistic and potentially pre-clinical assessment. The SAR study on divamide A and its congeners revealed that the tertiary structure bestowed by the cyclic lysinoalanine residue is apparently necessary for the antiviral activity of the peptide to block HIV infection [33,39].

## 6. Phaeornamide: A Recent Lipopeptide-Derived Selidamide

Very recent work by the Piel group has revealed a new class of RiPP-derived lipopeptides, the selidamides [40]. This class is characterized by fatty acyl moieties attached to the side of the chain of (hydroxy)ornithine or Lys, respectively. This defining feature is catalyzed by the modifying enzymes of the GC5-related N-acetyltransferase (GNAT) superfamily [59,60]. Compared to non-ribosomal lipopeptides that are usually synthesized as congener mixtures, the selidamides are specifically fatty acylated with a fixed-length fatty acyl unit [40].

In addition to landornamide A [61] and spliceotides [62] that were discovered by proteusin mining, the continued further large-scale and targeted bioinformatic investigations for the additional proteusin pathway encoding GNAT in the genome of an arctic marine-derived α-proteobacterium, *Pseudophaebacter articus* DSM23566, led to the characterization of the selidamide phaeornamide. The structure of phaeornamide has been elucidated by a multifaceted approach employing a combination of chemical derivatization, labelling studies, and peptide deconstruction, where the key features are based on NMR determinations. Phaeornamide contains a MeLan ring that is acylated at 4-(*S*)-hydroxyornithine with 3-(*S*)-hydroxydecanoic acid (C10) fatty acid from primary metabolism [40].

**Figure 7:**
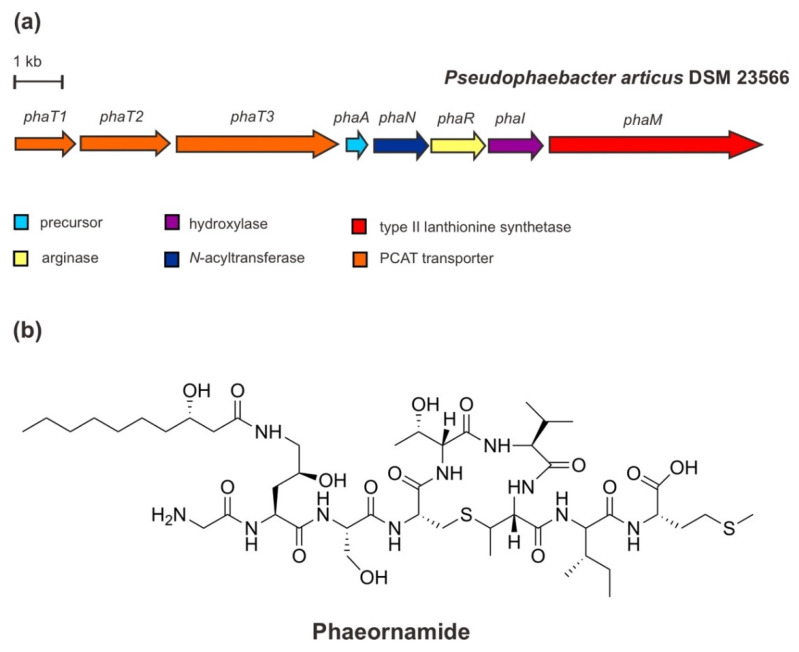
(**a**) Schematic architecture of the biosynthetic gene cluster of phaeornamide and (**b**) the chemical structure of phaeornamide.

The cluster genes for the phaeornamide from *P. articus* (Figure 7) comprises a precursor gene *phaA* encoding a PhaA NHLP-family precursor peptide, a *phaR* gene encoding an OspR-like peptide arginase, a *phaM* gene encoding a type II lanthionine synthetase, a *phaI* encoding a putative Fe(II)/α-ketoglutarate-dependent hydroxylase, a *phaN* encoding a GNAT N-acyltransferase, and dual-function transporter genes *phaT1/T2/T3*. Based on heterologous expression reconstruction, co-expression, and mutagenesis experiments, the biosynthesis of phaeornamide has been suggested in the following order: the precursor peptide is firstly modified by an ornithine formation from arginine (Arg) via loss of urea catalyzed by PhaR, followed by a hydroxylation of ornithine residue generating 4(S)-hydroxy-2(*S*)-ornithine catalyzed by PhaI, and subsequent acylation of (hydroxy) for fatty acid attachment catalyzed by PhaN GNAT. The installation of the MeLan ring by PhaM presumed to be independent of all other PTMs to some extent since corresponding intermediates could be observed at every step of the arginine processing event [39]. Moreover, it seems that the putative peptidase-containing ATP binding transporter PhaT is involved in the proteolytic and release step processes [63]. Apparently, PhaT cleaves at a specific conserved double glycine (Gly)—Gly-Gly—motif that is present in the sequence of the precursor PhaA, at the leader-core junction [40].

Peptide lipidation, such as acylation, can enhance stability, reduce polarity, and facilitate membrane interactions. Thereby, many peptides have been converted into drug leads or drugs by lipidation [64,65,66]. In addition to the therapeutic applications, lipidated peptides have also played an important role in biotechnological applications (e.g., biosurfactants), especially due to their ecological roles related to their primary functions to survive under unfavorable conditions. These functions include motility for growth and reproduction, antimicrobial activity to inhibit competitors, biofilm formations, and heavy metal chelation [67,68]. Interestingly, selidamides have demonstrated several similar structural properties [40]—particularly primary metabolism-derived fatty acid moieties and cyclic structure—with other classical and high medical importance non-ribosomal lipopeptides, such as anti-infectives daptomycin and echinocandins [69]. The PTM lipidation in RiPPs is still very rare. To date, the other reported lipopeptide RiPPs are only the lipolanthines [70] and goadvionins [71] which hybridize with polyketides, and the cyanobactins containing prenyl moieties [72].

## 7. A Diphosphorylated RiPP of Phospeptin

In addition to bacteria—especially uncultivated bacteria—from marine macroorganisms, such as sponges and tunicates, the biosynthetic potential of the global microbiomes from seawater—particularly the deep-community, also offers more promising novel enzymology as well as natural products since they remain widely underexplored. Through larger genomics assembly of the metagenome-assembled genomes (MAGs) and single-amplified genomes (SAGs), Paoli et al. (2022) [41] have lately identified a lineage rich in BGCs of uncultivated bacteria *Candidatus* Eudoremicrobiaceae (e.g., *Ca*. E. malaspinii) and characterized two novel RiPP natural product pathways. One of them is proposed to be named phospeptin, a novel diphosphorylated linear peptide.

**Figure 8:**
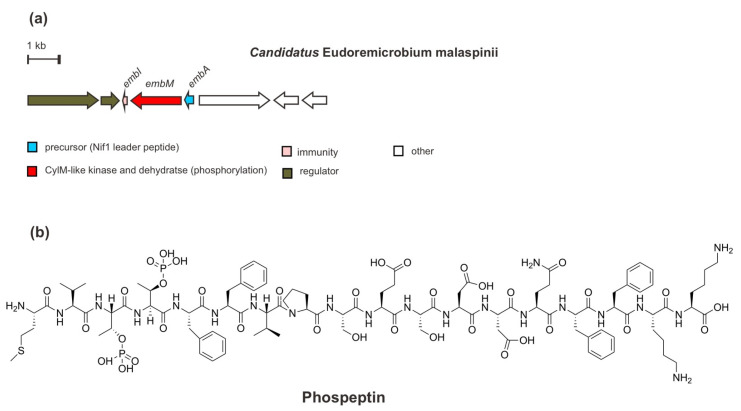
(**a**) Schematic architecture of biosynthetic gene cluster of phospeptin and (**b**) the chemical structure of phospeptin.

From the genome mining of the deep-seawater species *Ca*. E. malaspinii, a RiPP BGC has been found (Figure 8), which encodes a precursor peptide EmbA modified by only a PTM/maturase enzyme EmbM synthesizing phospeptin. Based on sequence similarity, the precursor peptide EmbA is predicted to be a class II lanthipeptide, containing an N-terminal leader peptide sequence homologous with that of the Nif11 (nitrogen fixation) enzyme and a C-terminal region of a typical Gly-Gly cleavage motif. Moreover, EmbM shows predicted similarity to protein kinases and to the dehydratase domain of a cytolysin CylM synthetase (type II lanthipeptide LanM synthetase) but lacks a zinc-dependent cyclization domain. In line with this finding, a Cys residue, which is required during macrocycle installation, is absent in the precursor peptide. Such a biosynthetic enzyme LanM-like synthetase without a cyclization domain is also found in polytheonamide dehydratase *PoyF*, as mentioned previously. Moreover, the functional analysis through heterologous co-expression and mutagenesis of EmbAM revealed that EmbM is responsible for the phosphorylation of threonine (Thr) residues in the precursor core peptide EmbA, Thr3 and Thr4, in agreement with an in silico study. However, the critical active residues that are in charge of catalyzing the elimination of phosphate are missing. Eventually, the diphosphorylated peptide is the terminal product of the EmbM PTM enzyme, as confirmed by MS^2^ and NMR analysis. Furthermore, EmbI, which was also included in the co-expression test, demonstrated its immunity role in equipping the native organism and probably the heterologous expression host with resistance to the biological activity of the cluster product. At the same time, other ORFs encoding putative proteins such as regulatory proteins for sensor histidine kinase and response regulator, glutamine aminotransferase for biosynthesis of guanosine nucleotides, and proteins involved in cell wall degradation or remodeling are also detected in the BGC.

Intriguingly, instead of antibacterial or cytotoxic activity, the phosphorylated form of active phospeptin exhibits a considerable human protease inhibitory activity toward neutrophile elastase (anti-inflammatory) at an IC_50_ of 14.3 µM) which is as good as fungal cyclopeptide natural products [73,74]. However, the ecological function of this peptide requires further study.

## 8. A Novel Proteusin Pathway for Pythonamide

**Figure 9:**
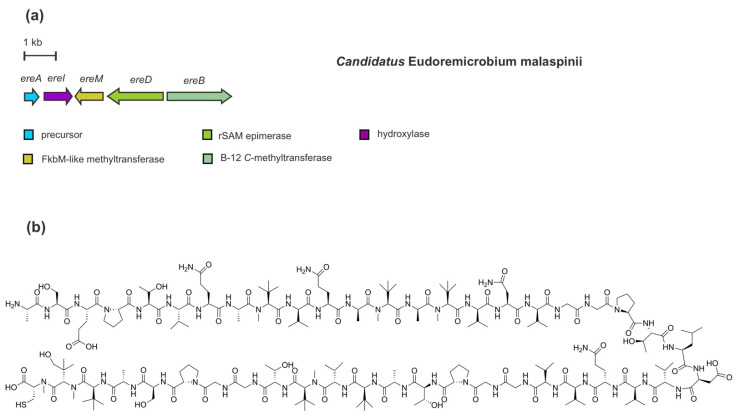
(**a**) Schematic architecture of a minimalistic biosynthetic gene cluster of pythonamide and (**b**) the chemical structure of pythonamide. Other genes (not shown) are presumably dispensable for in vitro heterologous expression of pythonamide biosynthetic cluster in *M. aerodenitrificans*.

Another recent microbiomics-driven RiPP pathway discovery, as mentioned above, is proposed to be called pythonamide [41]. In comparison to the polytheonamide’s BGC, this complex proteusin-predicted RiPP has been identified in a rather short BGC (5.2 kb) in *Ca.* E. malaspinii. The BGC of *ereAIMBD* (Figure 9) encodes an NHLP precursor peptide and four PTM enzymes which are responsible for the installation of up to 21 modifications, including hydroxylation, methylations, and epimerizations. In addition to the functional analysis through heterologous co-expression, the installations of these PTMs have also been corroborated by a combination of MS^2^ analysis, isotope labelling, and NMR elucidation [41].

The NHLP-like precursor peptide EreA contains 46 amino acids of the core peptide with mainly hydrophobic residues, including a four-repeated GGP(T/S) (Gly-Gly-Pro-Thr/Ser) motif and 16 valine (Val) residues. In addition, a cleavage motif of AVAGG (Ala-Val-Ala-Gly-Gly) is also present in the precursor peptide sequence. Among the PTM enzymes, EreM belongs to the FkbM-like SAM-dependent *O*-methyltransferase family. Interestingly, this enzyme catalyzes multiple N-methylations (up to six methylations) of the peptide backbone. Such methylation is the first report of N-methyltransferase activity by EreM, demonstrating a new function of FbkM family members in RiPP modification. So far, enzymatic N-methylation of a backbone amide bond—largely found in NRPS machinery—has specifically defined the RiPP class of borosins as represented by omphalotin A [31,75,76,77]. This modification plays an important role in modulating biological activity, selectivity, conformational structure, and pharmacokinetic properties of peptides [78]. Multiple methylations catalyzed by EreM have been observed in concurrence with EreI, indicating the interaction of EreM-EreI is a requisite for multiple amide bond N-methylations to happen to some extent. Encoded in the most upstream of other PTM-encoding genes, EreI (22% amino acids identity with *PoyI*) is a Fe(II)/α-ketoglutarate-dependent oxygenase belonging to the hydroxylase protein family. The hydroxylation most likely occurs at the C-terminal Val residue as observed in a *tert*-Leu methyl residue in the core peptide. In addition to peptide backbone N-methylations, a cobalamin B12 C-methyltransferase EreB catalyzes C-methylation of Val side chains. Thus, a *tert*-Leu methyl residue results from the addition of one methyl residue and incorporation of one oxygen. Of note, this functional analysis of EreB, however, has been only observed in *Microvirgula aerodenitrificans* (instead of *E. coli*), containing a complete pathway for B_12_ biosynthesis, through an engineered mutant Δaer *M. aerodenitrificans* heterologous co-expression system. For epimerization, rSAM epimerase EreD (36% amino acids identity with *PoyD*) catalyzes l-to-d epimerizations of five Val residues and two Ala residues [41].

## 9. Concluding Remarks

The exceptional metabolic capabilities of marine bacteria allow them to adapt and survive in almost all areas of the marine habitat. Yet, they remain only a partially explored reservoir of novel natural products and biosynthetic features. Bacterial secondary metabolite natural products are produced by devoted biosynthetic machineries, which are commonly encoded within a set of the gene cluster (a biosynthetic gene cluster, BGC) in the genome. After the structural identification of novel metabolite natural products, annotating and elucidating BGCs are necessary to understand and ultimately exploit the biosynthetic pathway. The discovery of RiPPs has taken full advantage of the knowledge of biosynthetic traits and enzymology aided by the advancements of genome sequencing technology and synthetic biology toward mutagenesis, heterologous expression, or pathway engineering [31]. Bioinformatics-guided genome sequencing and synthetic biology methods can identify a cryptic BGC, facilitating the production of modified or tailored gene-encoded RiPPs with minimalistic gene cluster sets [79]. Moreover, Carroll and his co-workers [80] also highlighted that in order to exploit unique marine microbial natural product diversity, genome sequencing is recommended to guide strain selection, followed by innovation in cultivation.

As mentioned above, recent discoveries of RiPPs from marine bacteria have demonstrated that some PTMs resemble those in non-ribosomal peptides, such as d-amino acids, amide N-methylations, side-chain cross-links, and fatty acid moiety/lipidation that have made the structural gap between these two classes of peptides indistinct. Lipidation in phaeornamide, which is very rare in RiPPs, may broaden the biosynthetic scope of ribosomal systems of the class of selidamides and allow new opportunities for the further biocatalytic toolbox of therapeutic peptide application. In addition to the RiPP origin of lipopeptide, however, ammosamides and lymphostin demonstrated a previously unknown overlap between RiPPs and pyrroloquinoline alkaloids. It is now clear that an RiPP also serves as the scaffold in the biosynthesis of ammosamides and lymphostin.

With the advances in genome sequencing, host mining or metagenomic strategies aided by the single-cell genomics methods [81,82], it has also become feasible to access other elusive RiPP natural products from uncultivated marine bacteria. They are exemplified by the discovery of polytheonamides from a sponge symbiont which were initially assumed non-ribosomal peptide synthetase products and divamide A from tunicate symbiont that successfully confirmed the predicted structure overcoming the limited resource supply. Furthermore, in addition to the microbiomes of marine invertebrates, the investigation of more complex microbiomes in the open ocean (seawater) at the global scale—that is reconstructed and integrated in Ocean Microbiomics Database (http://microbiomics.io/ocean/) [41,83]—led to the discovery of two novel RiPP pathways, phospeptin and pythonamide. Some divergences between bioinformatics predictions and biochemical and functional characterizations of PTM enzymes in these pathways have demonstrated both the boundary of functional reference through sequence homology as well as the necessity for continued experimental verifications. Thus, the synthetic biology enables further functional analysis and structure–activity relationship, leading to a platform for potential bioactive assessment, including a chemical template for the development of therapeutics or drug leads [84,85].

It is noteworthy to mention that the current global analysis of BGCs in marine bacteria genomes has revealed that RiPPs biosynthesis was the second most common BGC (21%), following terpenoids (24%) [86]. Interestingly, at the gene cluster family level of the global ocean microbiome investigation, the majority of novel diversity also consists of predicted RiPPs besides terpenoids. Surface or deeper sunlit waters and the least explored communities such as polar and deep habitat were associated with higher abundance of RiPPs BGCs [41]. However, of the aforementioned marine bacterial RiPPs—both from cultivated and uncultivated bacteria—not many have been experimentally characterized or their product investigated during the past decade. Thereby, many more uncharacterized BGCs still await to be realized and exploited. It is anticipated that the advancement in the discovery method through the genome-mining approach, engineering and synthetic biology strategies should aid marine bacterial RiPP to continue to expand for future applications.

## Figures and Tables

**Table 1:** Summary of recently identified marine bacterial RiPPs reported during 2012–2022.

RiPP	Class	Organism Origin	Biosynthetic Feature	Biological Function/Potential Application	Reference
Polytheonamides A and B	Proteusin	Uncultured bacterial symbiont of sponge *Theonella swinhoei* (*Entotheonella* factor)	Nitrile hydratase- like LP (NHLP)	Cytotoxic activity toward MCF-7 breast cancer cell line/ Anticancer agent	[37]
Ammosamides	Pearlin *	*Streptomyces* sp. CNR-698 (Deep sediment)	Peptide aminoacyl- tRNA ligase	Cytotoxic activity toward HCT-116 colon carcinoma cells/ Anticancer agent	[38]
Lymphostin	Pearlin *	*Streptomyces* sp. CNR-698; *Salinispora* sp.	Peptide aminoacyl- tRNA ligase	mTOR inhibitor/ Immunosuppressant	[38]
Divamide A	Cinnamycin-like Lanthipeptide	Uncultured symbiont of	Methyllanthionine, N-terminal trimethylation	Antiviral toward HIV-infected cells/anti-HIV agent	
		tunicate *Didemnum molle* (cyanobacterium *Prochloron didemni*)			[39]
Phaeornamide	Lipopepide- derived Selidamide *	*Pseudophaebacter* *articus* DSM23566 (Artic ocean)	Fatty acylation, lipidation	ND **	[40]
Phospeptin	ND **	*Candidatus* Eudoremicromium (*Ca*. E.) malaspinii (Deep seawater)	Di-phosphorylation	Neutrophil elastase inhibitor/anti- inflammatory agent	[41]
Pythonamide	ND **	*Ca.* E. malaspinii	FkbM-mediated peptide backbone N-methylation	ND **	[41]

* New class RiPPs; ** Not determined yet.

## Data Availability

Not applicable.
